# Gonorrhea as a Social Problem

**Published:** 1901-07

**Authors:** 

**Affiliations:** Atlanta, Ga.; Atlanta, Ga.


ATLANTA

### Journal-Record of Medicine.

Successor to Atlanta Medical and Surgical Journal, Established 1855, and Southern Medical Record, Established 1870.

VOL. III. JULY, 1901. No. 4.

BERNARD WOLFF, M.D., EDITOR, Nos. 319–20 Prudential.

Published Monthly.

M. B. HUTCHINS, M.D., BUSINESS MANAGER, No. 64 Marietta St.

### *ORIGINAL COMMUNICATIONS.*

### GONORRHEA AS A SOCIAL PROBLEM.*

BY DRS. CHILDS & CHAMPION,

ATLANTA, GA.

It can be stated without fear of successful contradiction that most of the diseased pelvic conditions of women and the untold suffering they have to endure are traceable to gonorrheal infection, and the blame can be laid in a large measure at the door of the medical profession.

In a report of the committee of the section of State Medicine of the American Medical Association, Humiston states : "Without classifying doubtful cases as gonorrhea, 90 per cent., at least, of pelvic inflammatory troubles are attributable to gonorrhea, the infection being usually of a mixed character—gonococcal with some one of the pyogenic micrococci ;" and Joseph Price says: "That in over one thousand sections for pelvic suppuration, 90 per cent. were

* Read at meeting Medical Association of Georgia, Augusta, April, 1901.

traceable to gonorrheal infection, and that 90 per cent. of these histories are reliable and clear." Czerny "regards it as impossible to tell"; and Boldt estimates from 5 to 6 per cent., admitting it as a guess. Ten per cent. is the estimate of Skene, Robb, Eastman and Bovee; Wathen, "nearly all of the cases," and Mann, "nearly all of those who have not borne children." The estimate of Pozzi and Frederick is 75 per cent.

Naeggerath thirty-five years ago stated that a "vast proportion of the death-dealing diseases in women were due to gonorrhea which the husband had had years before." While he who makes a statement like this in regard to a disease that is accountable for so much human suffering may be branded as an extremist, if we will reflect for a moment upon the highly infectious nature of gonorrhea, how lightly it is looked upon by the laity and many members of our profession, the number of gonorrheal patients still uncured that marry, the number that are dismissed by the physician as cured, but time proves to the contrary—these patients being dismissed without an examination of the urethra for stricture, not even the urine for "clap-shreds" (the secretions, if any, should be obtained from the urethra, prostate, and seminal vesicles and examined microscopically), we can form some idea as to the number of women necessarily infected. If it was possible to examine the urethra of the husband of every woman suffering with inflammatory conditions of the uterus and adnexa, in the great majority of cases the source of infection could be traced to the husband's urethra.

The effects of gonorrhea are more serious and permanent in the female, and when it has invaded the deep glandular structures, the tubes, ovaries, peritoneal cavity, it is a question whether it is ever cured. The number of cases of gonorrheal ophthalmia, the large per cent. of the inmates of the blind asylums due to gonorrhea, the number of sterile marriages due to the disease, should alarm the laity and the profession more than an occasional epidemic of yellow fever, smallpox or bubonic plague.

Now, the question arises, is gonorrhea a curable disease?

All cases in the male can be cured if the patient will follow instructions and submit to necessary treatment, whether medical or surgical.

In the report of the committee from the American Medical Association referred to, it is stated by Lydston that gonorrhea is curable "in the majority of cases"; Chismore, Fuller, and Verhoogen, "in the great majority"; Andry, "if the physician attends to it diligently and the patient is willing to care for himself as required and for as long a time as is necessary"; Feleki, "only a small percentage must, in consequence of the uncertainty of the duration of their infectiousness, or in consequence of serious complications, be looked upon as incurable. I estimate this proportion as about 3 per cent. Fingen states that "all are curable." The question is frequently asked as to the curability of anterior and posterior urethritis. In any case of gonorrhea that has covered a period of two weeks the posterior urethra is involved; which can be demonstrated by washing out the anterior urethra and having the patient to urinate in a glass, when "clap-shreds" will be seen. So, practically, in every case, of the disease the posterior urethra is involved. In our opinion it is curable either in anterior or posteria urethra.

We can state to a patient he is cured when he has no untoward symptoms, such as too frequent urination, getting up at night to urinate, pains in the urethra or bladder; when the urethra is free from stricture; when the prostate is not sensitive or enlarged; when the seminal vesicles are normal; and when, after repeated examinations, the gonococcus is not found and the urine is free from pus cells. When we find a patient's urethra in the condition as described it will be safe for him to marry, and if he contemplates such a step at the time, not to wait three, six, or twelve months to determine whether he is well or not, because he will in the meantime, in all probability, contract the disease again.

Within the past two years it has been our rule to stain and examine the discharge from every urethra we have treated. Frequently patients present themselves for treatment when there are only "clap-shreds" present. When such is the case, we centrifuge the urine, collect the sediment, stain and examine it for the gonococcus. If necessary, we throw a strong solution of silver nitrate into the urethra so as to produce some discharge for examination. We have 219 specimens stained, mounted with name and date, and whether acute or chronic, obtained from patients in private practice, and in 98 per cent. of examinations made where discharge was collected from the meatus for examination, whether acute or chronic form that had persisted for years, the microscope showed the gonococcus present. In other words, when there is a discharge of pus from the urethra 98 per cent. of the cases will prove to be infectious. Of the 219 specimens obtained, 43 were from married men living with their wives. Of the 43 married men 21 voluntarily stated that their wives were in bad health and were being treated for some female trouble. No statement was made by the other 22 or questions asked in regard to the health of their wives. It is a notorious fact that when a married man comes for treatment for gonorrhea, or any urethral trouble due to the disease, that he states his wife is an invalid and tries to excuse his sins on account of his wife's health being wrecked, which he alone is accountable for.

Something should be done to check the ravages of this disease, which is more serious and far-reaching than any disease that affects womankind. Regular inspection and examination of inmates of houses of prostitution, as practiced in European countries, have been suggested; the examination by law of the candidates for matrimony has been advocated and other legal methods suggested, but I think it will be admitted that regulation by statute is impracticable.

The only feasible way to check the ravages of gonorrhea is through the medical profession. Any law instituted or put into practice will get into the hands of politicians, or incompetent men, who will use the office for self alone, and be an injury to the profession and the public generally. Good can be accomplished and results obtained by teachers in the medical schools indelibly impressing upon the minds of the men that will make up the profession of the future the serious nature of gonorrhea and its far-reaching evil consequences. Let each individual member of the profession take and feel enough interest in the gentler sex to see to it that every patient that we treat with gonorrhea has a urethra that is beyond a doubt free from gonococci before he is dismissed as cured or allowed to marry. It should be the duty of every man who undertakes to treat the disease to first impress upon the patient the importance of always having the care and attention of a competent physician, and not a druggist to tide him over a disease that has such serious consequences. The patient should always be impressed that so long as " clap-shreds " or pus can be found in the urine, that the urethra is still diseased, and in all probability infection is possible. It too frequently happens that men with a slight discharge from the urethra tell us they consider the discharge harmless, not knowing that the slight morning drop is teeming with thousands of gonococci.

As stated by König: " No doubt it will be always impossible to get rid of this disease entirely, but if it were generally known how serious may be the consequences, the number of infections would no doubt be much less. The education of the public in this respect is chiefly in the hands of the medical profession. Have we been doing our duty in this respect? "

*Prudential Building.*

